# Supplementary material for: Endoplasmic reticulum oxidoreductin provides resilience against reductive stress and hypoxic conditions by mediating luminal redox dynamics
Source: Plant Cell. 2022 Jul 12;34(10):4007–27. doi: 10.1093/plcell/koac202 (PMC9516139; doi:10.1093/plcell/koac202)
Supplement: koac202_Supplementary_Data [file TPC2021RA01165R1_Peer Review Report.pdf › koac202_Supplementary_Data/TPC2021RA01165R1_Peer Review Report.pdf]

Endoplasmic reticulum oxidoreductin provides resilience against reductive stress and hypoxic conditions by mediating luminal redox dynamics

José Manuel Ugalde, Isabel Aller, Lika Kudrjasova, Romy R. Schmidt, Michelle Schlößer, Maria Homagk, Philippe Fuchs, Sophie Lichtenauer, Markus Schwarzländer, Stefanie J. Müller-Schüssele, Andreas J. Meyer

Corresponding author: Andreas J. Meyer andreas.meyer@uni-bonn.de

Review timeline:

**TPC2021-RA-01165**	Submission received:	Jan. 4, 2022
	1st Decision:	Feb. 14, 2022 *revision requested*
**TPC2021-RA-01165R1**	1st Revision received:	Apr. 18, 2022
	2nd Decision:	May 31, 2022 *acceptance pending, sent to science editor*
	Final acceptance:	July 5, 2022

REPORT: (The report shows the major requests for revision and author responses. Minor comments for revision and miscellaneous correspondence are not included. The original format may not be reflected in this compilation, but the reviewer comments and author responses are not edited, except to correct minor typographical or spelling errors that could be a source of ambiguity.)

TPC2021-RA-01165 1st Editorial decision – *revision requested* **Date**

Thank you for submitting your best work to The Plant Cell. The editorial board agrees that the work you describe is substantive, falls within the scope of the journal, and may become acceptable for publication, pending revision and potential re-review.

The reviewers are in agreement that the findings are exciting and make a substantial contribution to the field. However, they propose a number of editorial changes and request further explanation. They also request additional experiments so we ask you to pay attention to the following points in preparing your revision:

1) Provide phenotypic analyses of multiple ero mutant lines and amiRERO2 transgenics.

2) Provide primary root growth measurement for experiments shown in Fig 2G.

3) Ensure the same ero1 alleles are used in all panels of Fig 6.

-- Reviewer comments:

Reviewer #1 (Comments for the Author):

This manuscript describes studies that were designed to explore the role of the endoplasmic reticulum oxidoreductins (ERO)1 and 2 in oxidative protein folding in the ER. Data are presented from only one ero1, one ero2 and one ero/ero2 double mutant line. This is a limitation to data interpretation but perhaps not a major obstacle. The conclusions can only be drawn from the analysis of these mutants. Nevertheless, the study is elegant and the data are convincing. The results show that the ero1ero2 double mutants have no phenotype under optimal growth conditions but they show increased sensitivity to reductive stress and hypoxia. This manuscript is extremely well written and the discussion is particularly impressive, since give an objective an analysis of the data and its implications. Clearly the ERO proteins function alongside other systems that serve to regulate the redox state of the ER.

Reviewer #2 (Comments for the Author):

The manuscript by Ugalde et al. characterizes the functions of the ER oxidoreductins (ERO1 and ERO2) in Arabidopsis. As a double knock-out ero1 ero2 was known to be lethal, the authors use a microRNA strategy to partially inactivate the ERO2 gene in the context of the ero1 mutant. This led to the isolation of a viable ero1 ero2

double mutant which is highly sensitive to reductive stress and hypoxia. Then, they use an elegant strategy, a glutathione redox potential sensor roGFP2 to first demonstrate that ERO1 and ERO2 are type II ER membrane proteins, and then, to characterize the ER redox dynamics in vivo in the context of wild-type and ero1 ero2 mutant plants. Overall, Ugalde et al. present a new and original work addressing the central role of EROs in regulating the ER redox homeostasis under reductive stress and hypoxia conditions. In general, the manuscript is very clearly written and the presented experiments support major conclusions. The following remarks have to be considered:

1) To characterize a viable ero1 ero2 mutant, the authors isolated different amiRERO2 lines in wild-type and ero1 mutant backgrounds. While the molecular characterization of these different lines is well performed, the authors decided to present phenotypic data on only one line (#5). To support the fact that all the study is performed on this particular line, it would have been accurate to show the phenotypes (at least the reductive stress sensitivity) of other isolated lines. In the same way, as the ero1-4 mutant looks to be weak allele (Suppl fig1), have the authors tried to obtain a viable ero1-4 ero2?

2) Concerning flooding experiments (fig 2), plants were submerged in the dark. Is there any rationate to perform this treatment in the dark, which looks not really physiological? Also on figure 2, the authors choose to show the impact of hypoxia on the rate of root tip regrowth. This criteria is quite atypical to quantify the impact of stress on root growth. Primary root growth measurement, as in figure 1 should also be represented.

3) To target the Grx1-roGFP2iL to the ER, the authors should indicate why they use the chitinase TP in addition to the HDEL sequence. In these experiments, it is also not justified why the ER glutathione redox potential is measured in hypocotyl cells of 5-day old plantlets (figure 4) as in other experiments, roGFP redox ratios are monitored in root elongation zone cells of plantlets (figure 5) or leaf disks of 4-week old plants (figure 6-7). May the heterogeneity of the analysed tissues influence the conclusions?

4) Line 361, the authors suggest that the ERO activity is expected to stop after the sharp drop in O2 supply. I do not understand why the CHX experiment is designed to answer to this question. Is this hypothesis supported by other data? Also, the CHX treatment which has a wide-range protein translation inhibition effect is quite difficult to interpret, including the massive roGFP oxidation in the mutant.

5) Figure 6 A indicates ero1-3, as 6B and D indicate ero1-7.

TPC2021-RA-01165R1 1st Revision received April 18, 2022

Reviewer comments and **author responses**:

We are grateful to both reviewers and the editors for the very positive and encouraging feedback. We revised the manuscript accordingly and specifically addressed the three following points highlighted by the editors:

1) Provide phenotypic analyses of multiple ero mutant lines and amiRERO2 transgenics.

Null alleles for *ero1* end *ero2* had been described before (Fan et al., 2019, Plant Physiol. 180, 2022-2033; www.plantphysiol.org/cgi/doi/10.1104/pp.19.00020) and thus we have deliberately not aimed to select multiple null alleles again for this work. For *ero1*, however, the allele *ero1-4* with residual expression (Supplemental Figure S1) is a functional null mutant for ERO1 unequivocally displayed by its pronounced sensitivity to DTT. This sensitivity is very similar to the sensitivity observed for *ero1-3* that had been previously described (new Supplemental Figure S2).

The selection of suitable mutants for further physiological characterization from phenotypic prescreens is now more transparently documented in a new supplemental figure (Supplemental Figure S5). Specifically, we show the DTT-sensitivity of three independent amiR*ERO2* transgenics and three independent *ero1 ero2* lines. Although for amiR*ERO2* line #5 initially had shown the lowest residual *ERO2* transcript abundance (Supplemental Figure S3B), the lines #16 and #17 showed a more pronounced DTT-sensitivity (Supplemental Figure S5A). The increased DTT-sensitivity in these lines may be explained by the sum of both ERO isoforms. The slightly higher transcript abundance for *ERO1* in line amiR*ERO2#5* may partially compensate for the loss of ERO2 activity. However, these knockdown lines for only *ERO2* were of no specific interest in the context of this work, where they were only a first step on the way to the double mutants. Similarly, DTT-sensitivity was analyzed for different independent *ero1 ero2*

lines (Supplemental Figure S5B). While all these lines showed pronounced sensitivity, the most severely affected line, *ero1 ero2#5,* was selected to be equipped with the Grx1-roGFP2iL sensor and further physiological characterization.

2) Provide primary root growth measurement for experiments shown in Fig 2G.

We have revised Fig. 2 to better present the root growth phenotypes of wild-type and *ero1 ero2* double mutants after hypoxia. Revised Fig. 2F shows images of the seedlings on agar plates either as controls grown continuously under normal atmospheric conditions or seedlings that were exposed to hypoxia for 3 hours followed by reoxygenation through ambient atmospheric conditions. Fig. 2G shows the quantitative analysis of the root growth for those seedlings that did resume growth. In wild-type and mutant seedlings that restarted root growth after hypoxia, the growth speed was indistinguishable between genotypes. It is noted though that the growth response was rather binary, i.e. an all-or-nothing response, where the *ero* double mutants showed higher rates of growth arrest than wild-type roots. This analysis is now included as the new Fig. 2H.

3) Ensure the same ero1 alleles are used in all panels of Fig 6.

The different labels resulted from renaming our original allele *ero1-7* to *ero1-3* to harmonize the nomenclature with that introduced by Fan et al. (2019). *ero1-7* in panels B and D was indeed *ero1-3* and this editing mistake has now been corrected. We apologize for the confusion caused by our oversight.

In addition, we provide a point-by-point response to all further reviewer comments below. All changes can be traced in the enclosed Word document in editing mode.

Reviewer #1 (Comments for the Author):

This manuscript describes studies that were designed to explore the role of the endoplasmic reticulum oxidoreductins (ERO)1 and 2 in oxidative protein folding in the ER. Data are presented from only one ero1, one ero2 and one ero/ero2 double mutant line. This is a limitation to data interpretation but perhaps not a major obstacle. The conclusions can only be drawn from the analysis of these mutants. Nevertheless, the study is elegant and the data are convincing. The results show that the ero1ero2 double mutants have no phenotype under optimal growth conditions but they show increased sensitivity to reductive stress and hypoxia. This manuscript is extremely well written and the discussion is particularly impressive, since give an objective an analysis of the data and its implications. Clearly the ERO proteins function alongside other systems that serve to regulate the redox state of the ER.

We are grateful to reviewer #1 for these very positive comments. We share the view that using only individual genetic lines can be a limitation. Yet, different T-DNA insertional lines for *ERO1* (*ero1-2* and *ero1-3*) and *ERO2* (*ero2-1* and *ero2-2*) have already been reported (Fan et al., 2019). The phenotypes upon DTT exposure we present in this report are consistent with those reported by Fan et al. for the different independent lines. Although *ero1-4* contains residual transcript, its DTT-sensitivity, which is similar to *ero1-3* (new Supplemental Figure S2) suggests that the mutant is severely constraint in ERO1 capacity and very likely lacks ERO1 function completely. For this reason, the *ero1-4* mutant was not considered any further. For *ERO2*, we did generate a series of *ero2* knockdown lines and a series of *ero1-3* amiR*ERO2* double lines (denominated as *ero1 ero2*). Information on these mutants including phenotypic characterization for consistently severe DTT-sensitivity is now included as a new supplemental figure (Supplemental Figure S5).

Reviewer #2 (Comments for the Author):

The manuscript by Ugalde et al. characterizes the functions of the ER oxidoreductins (ERO1 and ERO2) in Arabidopsis. As a double knock-out ero1 ero2 was known to be lethal, the authors use a microRNA strategy to partially inactivate the ERO2 gene in the context of the ero1 mutant. This led to the isolation of a viable ero1 ero2 double mutant which is highly sensitive to reductive stress and hypoxia. Then, they use an elegant strategy, a glutathione redox potential sensor roGFP2 to first demonstrate that ERO1 and ERO2 are type II ER membrane proteins, and then, to characterize the ER redox dynamics in vivo in the context of wild-type and ero1 ero2 mutant plants. Overall, Ugalde et al. present a new and original work addressing the central role of EROs in regulating the ER redox homeostasis under reductive stress and hypoxia conditions. In general, the manuscript is very clearly written and the presented experiments support major conclusions.

We thank reviewer #2 for kindly providing thoughtful and very helpful feedback on our manuscript. We answer the comments one-by-one below.

The following remarks have to be considered:

1) To characterize a viable ero1 ero2 mutant, the authors isolated different amiRERO2 lines in wildtype and ero1 mutant backgrounds. While the molecular characterization of these different lines is well performed, the authors decided to present phenotypic data on only one line (#5). To support the fact that all the study is performed on this particular line, it would have been accurate to show the phenotypes (at least the reductive stress sensitivity) of other isolated lines. In the same way, as the ero1-4 mutant looks to be weak allele (Suppl fig1), have the authors tried to obtain a viable ero1-4 ero2?

As indicated in our response to reviewer #1, we now added further data on the screening of multiple *ero1 ero2* double mutants (new Supplemental Figure S5). We appreciate the suggestion on generating a new *ero1-4 ero2-1* double mutant that could still be viable if *ero1-4* is indeed only a knockdown mutant. As detailed in our response to reviewer #1 above, the pronounced DTT-sensitivity of this mutant strongly suggests that *ero1-4* is a functional null mutant for ERO1 function, similar to *ero1-3*. Based on these observations there is no reasonable expectation for gaining additional information from this mutant or any derived crosses for the scope of our current work. Therefore, the *ero1-4* allele was not considered for further work here. However, additional crosses have been initiated for future work but will require several months for segregation and detailed analysis.

2) Concerning flooding experiments (fig 2), plants were submerged in the dark. Is there any rationate to perform this treatment in the dark, which looks not really physiological? Also on figure 2, the authors choose to show the impact of hypoxia on the rate of root tip regrowth. This criteria is quite atypical to quantify the impact of stress on root growth. Primary root growth measurement, as in figure 1 should also be represented.

Submerged plants were intentionally kept in the dark to prevent any photosynthetic activity that would otherwise lead to the formation of oxygen (and reoxygenation), which would counter the intended hypoxic conditions. Submergence in the dark is an experimental standard model when focusing on the hypoxia component of submergence stress. By contrast, submergence experiments in the dark-light cycle provide a distinct stress regime switching between hypoxia and hyperoxia. To make our rationale clear to the reader, we now modified the text in line 749. In the revised manuscript this reads now "*…to prevent all photosynthetic activity and hence endogenous oxygen formation that would lead to re-oxygenation and counter the intended hypoxic conditions.*"

We revised Fig. 2 to better present the hypoxia-induced phenotypes of seedlings and to quantify the effect of hypoxia on root growth (panels F-H). We observed the growth of roots after the hypoxic phase to be an all-or-nothing response. If the primary root meristem regains its activity after a 3 h hypoxic phase, resumed primary root growth was as fast as in WT seedlings. Yet, we observed that the number of roots resuming growth was lower for *ero1 ero2* than for the WT.

3) To target the Grx1-roGFP2iL to the ER, the authors should indicate why they use the chitinase TP in addition to the HDEL sequence.

The chitinase TP is routinely used to facilitate the import of recombinant protein constructs into the ER (via SRP). The C-terminal HDEL acts as ER retrieval signal, i.e. it is essential to prevent the ER resident protein from being passed down the secretory pathway. To avoid any misunderstanding, we now explain the function of both motifs more clearly in the Materials and Methods section (line 684). The text now reads as follows: *"...chitinase target peptide for mediating entry into the ER and the C-terminal -HDEL motif for ER retrieval."*

In these experiments, it is also not justified why the ER glutathione redox potential is measured in hypocotyl cells of 5-day old plantlets (figure 4) as in other experiments, roGFP redox ratios are monitored in root elongation zone cells of plantlets (figure 5) or leaf disks of 4-week old plants (figure 6-7). May the heterogeneity of the analysed tissues influence the conclusions?

It is correct that we have used different cell types, tissues or even whole seedlings in different experiments. The developmental stage of plants exposed to low oxygen conditions is critical for the extent of damage caused by hypoxia (Le Gac and Laux, 2019, Mol Plant, 12, 1422-1424, doi: 10.1016/j.molp.2019.10.004; Leon *et al.*, 2021, J Exp Bot, 72, 5841-5856, doi: 10.1093/jxb/eraa591). We now added a side-by-side comparison of the hypoxia-induced

changes in E_{GSH} in both young seedlings (7 days after germination) and disks from mature leaves (4-week-old plants). The data support the assumption that the general response to hypoxia is consistent in both systems (Supplemental Figure S12). It is apparent though that the signal-to-noise level is significantly better in leaf disks and thus leaf disks are the preferred experimental system for time course experiments. The explanation for the use of each tissue is now more clearly explained in L.298 for roots, L.273 for hypocotyl cells and L.335 for leaf disks.

Hypocotyl cells were used to image ER networks in steady conditions based on their strong sensor fluorescence signal and favorable signal-to-noise ratio. To illustrate this, we now added a detailed comparison of the sensor signal in cotyledon, hypocotyl, root and root tip (new Supplemental Figure S9). The permeability of hypocotyl epidermal cells, however, is severely restricted as emphasized by the fact that 10 mM DTT and 100 mM H_2O_2 had to be used to drive the sensor protein to its extreme ratio values for calibration. Therefore, epidermal cells from the root elongation zone were used in perfusion experiments because these cells are easily accessible to perfusion with external stimuli like DTT and H_2O_2 at much lower concentration.

4) Line 361, the authors suggest that the ERO activity is expected to stop after the sharp drop in O2 supply. I do not understand why the CHX experiment is designed to answer to this question. Is this hypothesis supported by other data? Also, the CHX treatment which has a wide-range protein translation inhibition effect is quite difficult to interpret, including the massive roGFP oxidation in the mutant.

The initial observation was that hypoxia initially causes a reduction of Grx1-roGFP2iL in the ER, which indicates a successive reduction of the luminal glutathione pool (Fig. 6A). We hypothesized that this reduction results from continued import of nascent peptides in the absence of ERO-mediated oxidation. In the absence of any established inhibitor of SEC61 that may directly interfere with peptide import into the ER, inhibition of protein synthesis had been a way of testing this hypothesis. Because CHX is the most commonly used inhibitor of translation in eukaryotic cells, we tested the hypothesis with CHX. The results provide strong support for our hypothesis. Yet, we fully agree with reviewer #2 that general inhibition of translation will eventually affect a plethora of other proteins leading to unwanted side effects. From earlier experiments it is known that cells survive incubation with CHX for at least 6 hours and still show physiologically meaningful responses (*e.g.*, Meyer and Fricker, 2002, Plant Physiol. 130, 1927-1937; Doyle et al., 2010, J. Exp. Bot. 61, 473-482). Even if available, other inhibitors obviously would have similar side effects. An alternative possibility would be to inhibit transcription, *e.g.* with Actinomycin D, but this would inevitably have an at least as broad impact on many other processes.

5) Figure 6 A indicates ero1-3, as 6B and D indicate ero1-7.

Many thanks for spotting this typo. Shortly before submission we had to revise the nomenclature of our *ero1* mutant collection to bring it in line with the nomenclature of published alleles. In this way, our original *ero1-7* was renamed to *ero1-3* as published by Fan et al. (2019). To keep everything consistent, this has now also been corrected in the panels 6B and D.

TPC2021-RA-01165R1 2nd Editorial decision – *acceptance pending* May 31, 2022

We are pleased to inform you that your paper entitled "Endoplasmic reticulum oxidoreductin (ERO) provides resilience against reductive stress and hypoxic conditions by mediating luminal redox dynamics" has been accepted for publication in The Plant Cell, pending a final minor editorial review by journal staff. At this stage, your manuscript will be evaluated by a Science Editor with respect to its presentation of scientific content, compliance with journal policies, and presentation for a broad readership.

Reviewer #1 (Comments for the Author):

Appropriate revision has been undertaken to address the points that were raised.

Reviewer #2 (Comments for the Author):

The authors have perfectly answered to all my comments. The new presented data improved the manuscript very much. I have no further remarks on this nice work.

Final acceptance from Science Editor **July 5, 2022**
